# Assessment of Disease Activity in Small Bowel Crohn's Disease: Comparison between Endoscopy and Magnetic Resonance Enterography Using MRIA and Modified MRIA Score

**DOI:** 10.1155/2015/159641

**Published:** 2015-11-22

**Authors:** Arnaldo Scardapane, Annalisa Ambrosi, Emanuela Salinaro, Maria Elisabetta Mancini, Mariabeatrice Principi, Alfredo Di Leo, Filomenamila Lorusso, Amato Antonio Stabile Ianora, Giuseppe Angelelli

**Affiliations:** ^1^Section of Diagnostic Imaging, Interdisciplinary Department of Medicine, University of Bari “Aldo Moro”, Piazza Giulio Cesare 11, 70124 Bari, Italy; ^2^Section of Gastroenterology, Department of Emergency and Organ Transplantation, University of Bari “Aldo Moro”, Piazza Giulio Cesare 11, 70124 Bari, Italy

## Abstract

*Objectives*. To retrospectively compare the results of the MRIA (magnetic resonance index of activity) with a modified MRIA (mMRIA), which was calculated excluding from MRIA formula the data of relative contrast enhancement (RCE). *Materials and Methods*. MR-E and corresponding endoscopic records of 100 patients were reviewed. MRIA, mMRIA, and SES endoscopic index were calculated for all the patients. Namely, MRIA was calculated as follows: (1.5 × wall thickening + 0.02 × RCE + 5 × intramural edema + 10 × ulcers), while mMRIA was calculated with the modified formula (1.5 × wall thickening + 5 × intramural edema + 10 × ulcers). *Results*. Mean MRIA and mMRIA values were 19.3 and 17.68, respectively (*p* < 0.0001). A significant correlation (*p* < 0.0001) was observed between MRIA and mMRIA scores and between both MR indexes and SES (*p* < 0.0001). *Conclusions*. mMRIA was comparable to MRIA in the evaluation of disease activity in Crohn's disease.

## 1. Introduction

Crohn's disease (CD) is a chronic progressive inflammatory disorder of the entire alimentary tract, classically involving the terminal ileum: ileitis is observed in 90% of the patients with small-intestinal CD, who in turn constitute 30–40% of all CD patients [1].

The disease is characterized by a relapsing and remitting course (flare-ups followed by clinical remission), posing the problem of repeated follow-ups over time for the assessment of disease activity.

To assess the severity of clinical disease activity, composite scores such as Crohn's Disease Activity Index (CDAI) or the Harvey-Bradshaw Index are used [2].

Ileocolonoscopy has been recognized as the gold standard for the evaluation of lesions in the colon and terminal ileum. To assess the severity of endoscopic inflammation, Crohn's Disease Endoscopic Index of Severity (CDEIS), the Simplified Endoscopy Score (SES-CD), or, in the postoperative setting, the Rutgeerts score was developed for use in clinical trials. However, there are several drawbacks related to the invasiveness, procedure-related discomfort, risk of bowel perforation, and relatively poor patient acceptance; moreover, it cannot always be complete in small bowel examination [2, 3]. Small bowel imaging, therefore, plays a vital role in diagnosing and phenotyping CD, thereafter assessing disease activity and complications [4]. MR enterography (MR-E) of the small bowel, thanks to the lack of ionizing radiation, along with very high soft-tissue contrast and multiplanar images has high diagnostic accuracy in the evaluation of luminal and extraluminal abnormalities [5].

A recent study, by proving evidence that the magnitude of quantitative MR changes (wall thickening, presence of edema and ulcers, and contrast signal intensity) closely parallels the severity of endoscopic lesions, allowed the creation of an MR index of activity (MRIA). The authors also demonstrated that MRIA is highly correlated with endoscopic scores [6].

In this paper, we have calculated a modified MRIA score (mMRIA) removing from the calculation suggested by Rimola et al. the data related to the relative contrast enhancement of bowel wall in order to correlate this score to MRIA and endoscopic score. Our purpose was to verify if this modified score, which is calculated from unenhanced images, might be used with the same accuracy compared to MRIA with possible advantages in pediatric patients, in the case of proven intolerance to gadolinium-based contrast and in subjects with severe renal failure and in repeated follow-up examination.

## 2. Materials and Methods

### 2.1. Study Population

During the period between March 2013 and March 2015, 100 patients aged between 16 and 81 were retrospectively enrolled in this study. Inclusion criteria were the following: (1) histologically proven Crohn's disease of the terminal ileum, (2) availability of a complete colonoscopy with terminal ileum exploration, (3) lack of surgical intervention related to Crohn's disease, and (4) MR-E performed within 60 days from endoscopy.

Disease duration ranged from 3 to 100 months (average 37 months). According to their treatment patients were divided into the following groups: (1) untreated patients (32/100), (2) patients assuming mesalazine (14/100), (3) patients assuming immunomodulators (31/100), and (4) patients assuming biological drugs (23/100). Our series was divided into two more groups related to the use of systemic corticosteroids (39/100 cases).

The informed consents of all the patients were available for both colonoscopy and MR-E. The study was approved by the Local Ethical Committee.

### 2.2. Endoscopic Evaluation

In this study, the endoscopic records of the 100 patients were reviewed. MR-E was performed between 4 and 60 days (mean 48 days) from endoscopy. In 28/100 cases it was performed before ileocolonoscopy, while in 72/100 patients MR-E followed endoscopy. Endoscopic exams were performed by a board certified gastroenterologist with 10 years of experience in performing ileocolonoscopy. For calculating the SES-CD, the intestine was divided into five segments: the ileum, the right colon, the transverse colon, the left colon, and the rectum. The degree of disease involvement in each of the five segments was determined by the assessment of four parameters: presence and size of ulcers (score 0–3), extent of ulcerated surface (score 0–3), extent of affected surface (score 0–3), and presence and type of narrowing (score 0–3) [7]. Each bowel segment may have values between 0 and 12 while the overall SES score is calculated as the sum of each segment's score and may range from 0 to 60. Both overall and terminal ileum scores were calculated in our series and disease activity categories were assessed according to the overall SES score (Table 1).

### 2.3. Magnetic Resonance Enterography Technique

All MR-E were performed on a 1.5-Tesla MR unit (Philips Achieva 1.5 T A-series, Koninklijke Philips Electronics N.V., Eindhoven, The Netherlands) with a 4-channel phased-array body coil in the prone position.

Patients were asked to take oral laxatives at variable times and personalized doses to cleanse the bowel and to fast an overnight before the exam.

On the day of the examination, 45–55 min before the MR, each patient received orally a solution of biphasic contrast medium, previously prepared by dissolving 250–300 mL of 18% mannitol in 1500 mL of water in order to achieve a 3.5%–4% solution.

Inhibition of bowel peristalsis was achieved by injecting 10 mL/mg N-butyl scopolamine (Buscopan, Boehringer Ingelheim, Florence, Italy) intramuscularly before starting the MR examination.

The imaging protocol consisted of the following breath-hold sequences (Table 2):

1. Coronal, axial, and sagittal 2D balanced turbo-field echo (BTFE): matrix 256 × 256; slice number 40; thickness 8 mm, with 4 mm overlap; shortest TE/TR; flip angle 90°; FOV 350–450; and acquisition time 21 s/sequence.
2. Coronal and axial T2W single shot turbo spin echo (SSh-TSE): thickness 4-5 mm; TE 100 ms; shortest TR; flip angle 90°; matrix 320 × 320; FOV 350–450 mm; and breath-hold acquisition.
3. Axial T2W single shot turbo spin echo SPAIR (SSh-TSE-SPAIR): thickness 4-5 mm; TE 100 ms; shortest TR; flip angle 90°; matrix 320 × 320; FOV 350–450 mm; and breath-hold acquisition.
4. Coronal T1w high-resolution isotropic volume (THRIVE): matrix 256 × 256; slice number 100; thickness 2 mm; SENSE factor 4; shortest TE/TR; flip angle 10; FOV 350–450; acquisition time 19 s/sequence; coronal and axial T1w high-resolution isotropic volume (THRIVE). This sequence was acquired before and after i.v. administration of 0.15 mL/Kg of gadolinium diethylene-triamine penta acetic acid (Gd-DTPA) 0.5 M followed by 20 of saline solution with a scan delay of 35, 70, and 120 seconds.
5. THRIVE acquisitions in the axial and sagittal plane: matrix 256 × 256; slice number 100; thickness 2 mm; SENSE factor 4; shortest TE/TR; flip angle 10; FOV 350–450; acquisition time 19 s/sequence.

### 2.4. Image and Statistical Analysis

Two radiologists experienced in abdominal MR imaging (AS with 10 years of experience and AASI with 15 years of experience identified throughout the paper as R1 and R2) who were unaware of endoscopic findings and results independently reviewed the examinations.

The analysis was performed using a dedicated postprocessing workstation DICOM viewer (OsiriX imaging software), examining the images from all sequences.

For all the examinations MRIA (magnetic resonance index of activity) and mMRIA (modified MRIA) of the terminal ileum were calculated by both the readers.

MRIA was calculated according to previous paper by Rimola et al. with the following formula:

 1.5 × wall thickness (mm) + 0.02 × RCE + 5 × edema + 10 × ulceration (where RCE corresponds to the relative contrast enhancement) [6].

The mMRIA was obtained by the same calculation, excluding the data related to relative contrast enhancement (0.02 × RCE), using the following formula: 1.5 × wall thickness (mm) + 5 × edema + 10 × ulceration.

**Figure 1:**
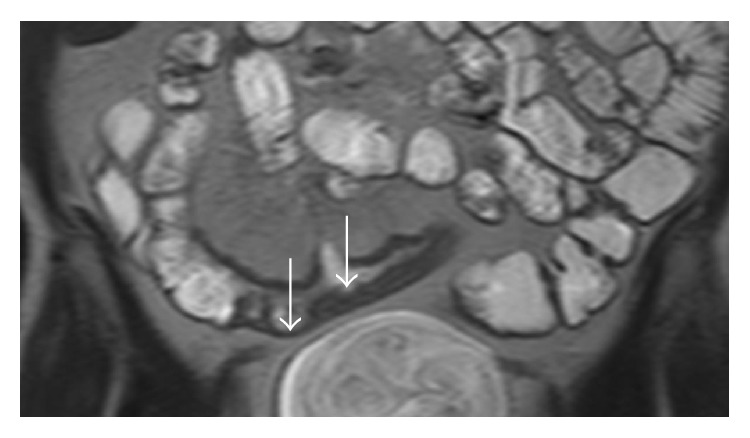
MRE, T2W coronal image. Ulcerations were diagnosed when irregular mucosal depressions were recognized within a thickened loop (arrow).

**Figure 2:**
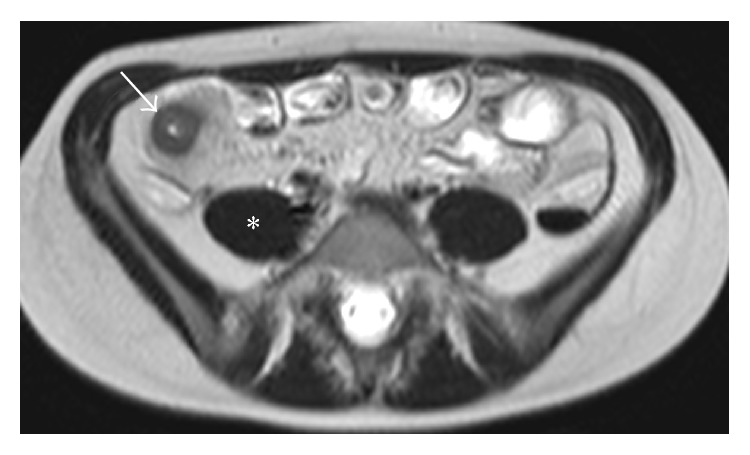
MRE, T2W axial image. Edema was diagnosed when the involved loops (arrow) showed higher signal intensity compared with psoas muscle (*∗*) in T2W images.

Quantitative measurements (wall thickness, RCE) were obtained from the most thickened loop. The presence of mucosal ulceration was defined as deep depressions in the mucosal surface within the thickened wall (Figure 1), and the presence of mural edema was defined as the high intensity signal on T2-weighted sequences relative to the psoas muscle's signal intensity (Figure 2).

Then relative contrast enhancement (RCE) was calculated according to the following formula: RCE = [(WSI postgadolinium − WSI pregadolinium)/(WSI pregadolinium)] × 100 × (SD noise pregadolinium/SD noise postgadolinium), where SD noise pregadolinium corresponds to the average of three SD of the signal intensity measured outside of the body before gadolinium injection, and SD noise postgadolinium corresponds to the SD of the same noise after gadolinium administration.

Cohen *K* test was used to calculate the interobserver agreement between R1 and R2 in recognizing qualitative MR findings such as wall ulceration and edema and to estimate agreement between endoscopy and MR-E. The quantitative evaluations such as wall thickness, RCE, MRIA, and mMRIA were compared between the two readers using the paired samples and two-sample Student's *t*-test, while the MRIA/mMRIA correlation was calculated using Pearson's correlation. The correlation between MRIA/mMRIA and endoscopic SES index was explored by Spearman's rank correlation. Statistic tests were performed using the software STATA/IC 14.

## 3. Results

Endoscopic evaluations and MR-E were considered adequate to the diagnosis in all the cases.

Endoscopy demonstrated the disease in all the patients. A terminal ileitis was diagnosed in 58/100 cases and ileocolitis in 23/100 patients, while Crohn's colitis was recognized in 19/100 patients. Overall SES ranged from 0 to 47 (mean 11.23, SD 8) while SES of terminal ileum ranged from 0 to 12 (mean 4.2; SD 3.3). On the basis of endoscopy a complete remission (score 0–2) was diagnosed in 3/100 patients (3%), a mild disease activity (score 3–6) in 27/100 (27%) cases, a moderate activity (7–15) in 51/100 (51%) patients, and a severe disease (>15) in the remaining 19/100 (19%) patients (Table 3). Both the readers demonstrated the involvement of the terminal ileum in 75/100 patients, while the terminal ileum was considered normal in 25/100 cases.

**Figure 3:**
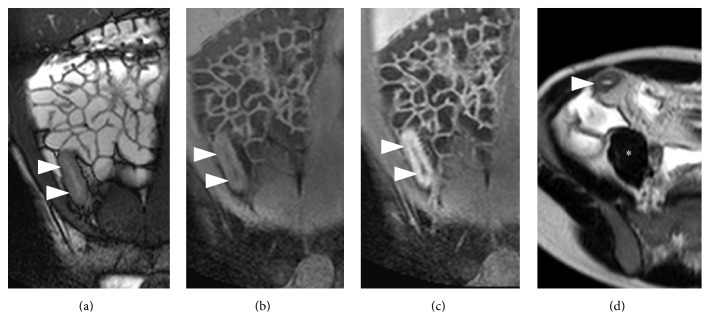
MR-E of a patient with terminal ileitis (ileal SES score 10; overall SES score 15). (a) Coronal B-TFE image. (b) Unenhanced coronal THRIVE image. (c) CE-coronal THRIVE. (d) Axial SSh-T2 image. MR-E shows a 10 mm thick, hyperenhancing, and ulcerated terminal ileum (arrowheads). T2 sequence demonstrates mural edema as terminal ileum (arrowheads) shows higher signal intensity than psoas muscle (*∗*). MRIA = 31.4; mMRIA = 30.

**Figure 4:**
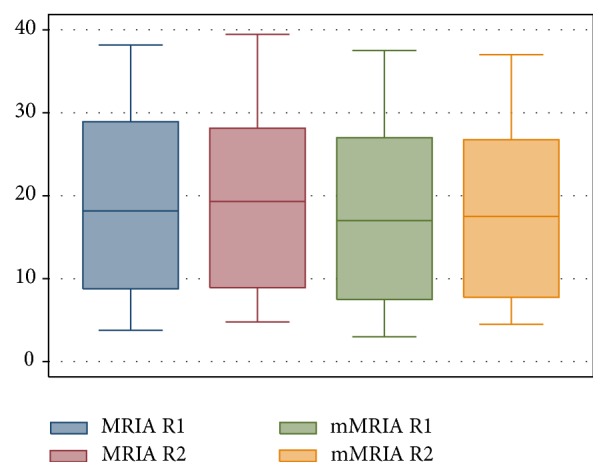
Box-plot of MRIA and mMRIA values of our series.

**Figure 5:**
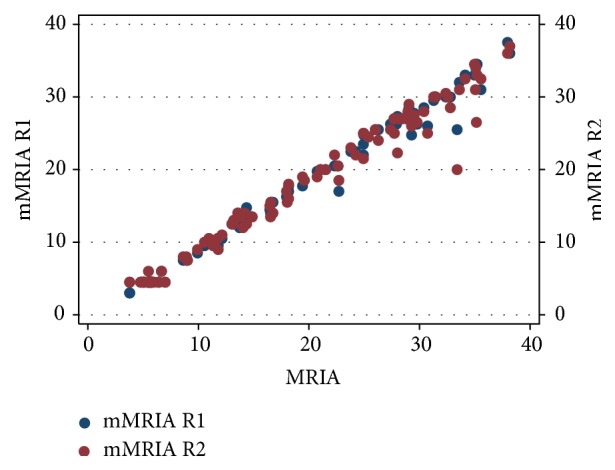
Scatter plot diagram demonstrating a strict correlation between MRIA and mMRIA values for both the reviewers.

Ulcerations were recognized in 52/100 patients by R1 and in 50/100 cases by R2 (*K* = 0.85) while mural oedema was diagnosed in 57/100 cases by R1 and in 60/100 by R2 (*K* = 0.83). Mean wall thickness was not statistically different between R1 (mean 7.44; SD 3.16) and R2 (mean 7.31; SD 3.2). MRIA and mMRIA indexes calculated on CE MR-E were not different between the two readers (*p* > 0.05); namely, MRIA ranged from 3.78 to 38.17 (mean 19.13; SD 10.68) for R1 and from 4.78 to 39.45 (mean 19.014; SD 10.47) for R2, while mean mMRIA ranged from 3 to 37.5 (mean 17.7; SD 10.38) for R1 and from 4.5 to 37 (mean 17.48; SD 10.07) for R2 (Figure 3). Paired samples *t*-test demonstrated a statistically significative difference between MRIA and mMRIA for both the readers (*p* < 0.0001) (Figure 4). In addition, MRIA and mMRIA showed a strong correlation between each other (Pearson's *r* = 0.99; *p* < 0.0001) (Figure 5).

**Figure 6:**
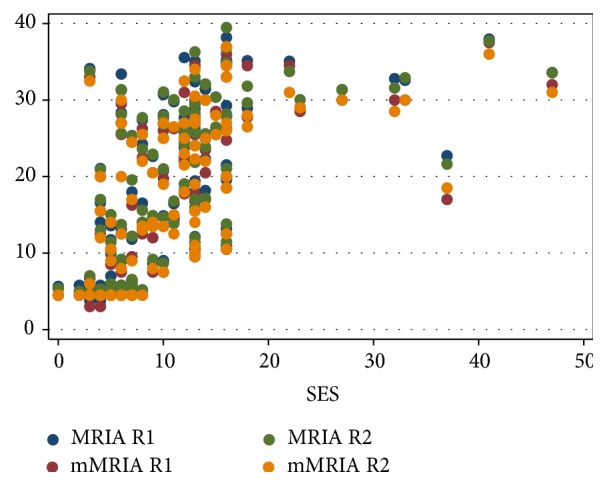
Scatter plot diagram of MRIA and mMRIA versus overall SES values.

**Figure 7 fig7:**
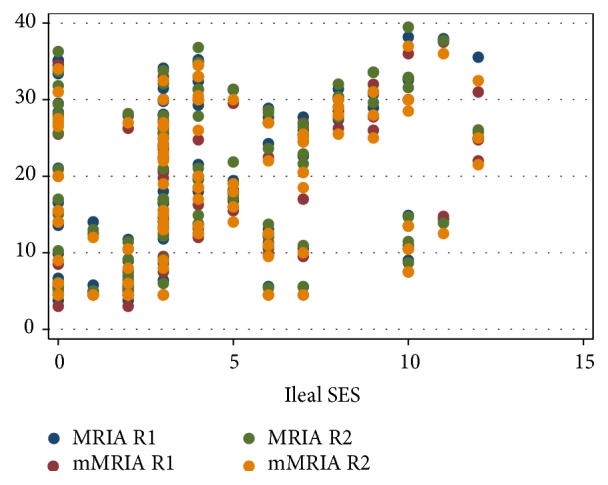
Scatter plot diagram of MRIA and mMRIA versus ileal SES values.

**Figure 8:**
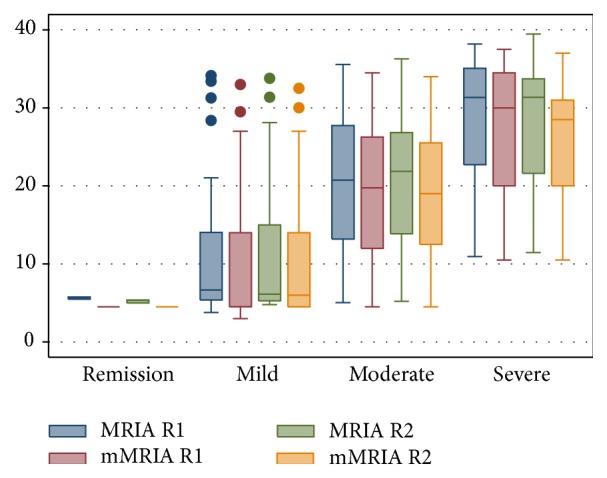
Box-plot diagram of MRIA and mMRIA values related to disease activity groups for both R1 and R2.

In our series, overall SES, ileal SES, and disease activity groups showed a strong correlation with MRIA and mMRIA. Correlation coefficients, which are summarized in Table 4, were statistically significative for R1 and R2 (Table 4, Figures 6 [Fig fig7]–8).

The one-way ANOVA comparing patients assuming different treatments showed significantly lower values of MRIA and mMRIA in patients without any treatment (*p* = 0.049), while no differences were found for subjects assuming mesalazine, immunomodulators, or biological drugs. In addition, MRIA and mMRIA values were significantly higher in patients using systemic corticosteroids for both the readers (R1: *p* = 0.018 and 0.035; R2: *p* = 0.016 and 0.033).

## 4. Discussion

In this study we compared the accuracy of MRIA to mMRIA which is calculated from unenhanced images only and we found that these two scores can be calculated with a very good interobserver agreement and have the same degree of correlation with the SES endoscopic score. To monitor disease activity and to guide appropriate treatment, CD patients require multiple imaging examinations repeatedly [8]. Cross-sectional techniques have advanced the ability to diagnose, classify, and monitor CD [9]; the desirable imaging modality would be one that is reproducible, free of ionizing radiation, and well tolerated. MR-E is a noninvasive technique not relying on ionizing radiation, showing high values of sensitivity and specificity in CD assessment [10] and nowadays the standard protocol is based on morphologic unenhanced images and on dynamic fast 3D spoiled gradient echo T1 fat-suppressed postcontrast sequence that evaluates the pattern of bowel wall enhancement [11].

Many studies stress the importance of evaluating the pattern of contrast enhancement of bowel wall, to assess disease activity [12]. Dambha et al., in a recent study in 2014, point out that intense mucosal enhancement postintravenous gadolinium is typical of active disease and that gadolinium administration identifies acute inflammatory change [13]. According to them, mucosal hyperenhancement has occasionally been found to be the only feature of recurrent disease in the absence of typical imaging findings [13]. Furthermore, in a study by Macarini et al., a significant reduction in wall thickness and contrast enhancement is considered the most reliable finding for predicting clinical remission in patients treated for active disease [10]. Other authors suggest assessing wall thickness and presence of edema in T2 sequence images as an initial global appraisal and only adding a basal and contrast enhanced T1 sequence in case an abnormality is detected [14]. Existing MRI activity scores generally include the evaluation of the pattern of contrast enhancement [6, 15–19]. Because the location of CD lesions in the intestine has a characteristic skip pattern, in which segments with severe ulcerative lesions can be adjacent to others with normal mucosa, Rimola et al. evaluated the MR findings associated with lesions of different endoscopic severity in a segment-by-segment analysis and also as a global MR index of activity (MRIA). MR changes found to be associated with disease activity and severity included edema, presence of ulcers, wall thickening, and relative contrast enhancement [6].

The aim of our paper was to compare MRIA to a modified index (mMRIA) calculated from unenhanced sequences. We found that mMRIA was statistically different from MRIA but it showed a very strong correlation with MRIA. In addition, mMRIA and MRIA demonstrated a comparable significative correlation with the endoscopic index of disease activity in the terminal ileum and with the disease activity calculated on the basis of SES score. As expected untreated patients (with clinical remission or mild disease activity) and patients using systemic corticosteroid (usually not responding to standard therapy) showed, respectively, lower and higher value of MRIA/mMRIA scores, while we did not find any difference among the remaining treatment groups. Probably the evaluation of multiple follow-up exams might put a light on the role of MR activity indexes in the evaluation of therapy efficacy. This experience shows that a reliable calculation of an MR based activity index can be achieved using unenhanced scans without any accuracy issue. However, despite these promising results, it should be remembered that the morphologic mural changes, the hyperintensity in T2-weighted sequences, and contrast enhancement are the expression different phenomena. Namely, T2-hyperintensity is due to mural edema, while contrast enhancement is related to wall hypervascularity and increased vascular permeability which frequently but not necessarily overlap [6, 17]. For this reason CE sequences and consequent patterns of mural contrast enhancement still play a crucial role in the assessment of Crohn's disease, namely, in the first assessment of the disease. On the other hand, RCE has a little effect on the MRIA calculation and it is not surprising that MRIA and mMRIA perform the same if compared with endoscopy as mMRIA actually constitutes the biggest part of MRIA score.

Our experience demonstrates that the calculation of a reliable index to assess Crohn's disease activity may be done with an unenhanced MR-E. According to these results, contrast injection could be avoided without concerns in patients needing repeated follow-up exams who generally show a better acceptance for unenhanced exams; furthermore, these findings can be really helpful with obvious advantages for pediatric patients and in case of renal failure or proven allergic reaction to Gd-based agents.

Our study has some limitations mostly related to its retrospective nature: firstly, our sample is inhomogeneous as patients had different disease durations and were following different treatments which might be responsible for anatomical changes we could not appreciate in a single MR exam. Secondly, the time slot between MR-E and endoscopy was variable leading to discrepancies between endoscopy and MR-E. Lastly, differing from previous studies, a complete per-segment and per-patient evaluation could not be done since the lack of a dedicate distension of the colon with oral contrast only allowed a reliable evaluation of the sole terminal ileum.

## 5. Conclusions

The results of this study show that the proposed mMRIA score provides a promising tool for assessing CD severity of the terminal ileum with a strong correlation with MRIA score and with endoscopy. The use of the mMRIA that is obtained from unenhanced images might be useful in different clinical settings such as follow-up exams, pediatric patients, and subjects with chronic renal failure or proven intolerance to Gd-based contrast media. Anyhow, further prospective studies are required to confirm mMRIA utility on both small bowel and colonic loops and to investigate its accuracy as a predictor of treatment efficacy in repeated studies.

## Figures and Tables

**Table 1:** Endoscopic activity of Crohn's disease according to SES scores.

Overall SES score	Crohn's disease activity
0–2	Disease in remission
3–6	Mild disease activity
7–15	Moderate disease activity
>15	Severe disease activity

**Table 2:** MRE sequences.

Sequence	Plane	Thickness/overlap	FOV	TR/TE (ms)	FA
Balanced turbo-field echo	Ax./Cor./Sag.	8/4 mm	350–450	Shortest/shortest	90°
T2 single shot	Ax./Cor.	4-5/0 mm	350–450	Shortest/100	90°
T1 high resolution isotropic volume (THRIVE)	Ax./Cor./Sag.	4/2 mm	350–450	Shortest/shortest	10°

**Table 3:** Disease activity of patients' series according to SES values.

SES group	Number of patients
Disease in remission	3
Mild disease activity	27
Moderate disease activity	51
Severe disease activity	19

**Table 4:** Correlation coefficients between MRIA, mMRIA, and endoscopic findings for R1 and R2.

	Overall SES	Ileal SES	Disease activity groups
MRIA R1	0.5965^*∗*^	0.3683^*∗*^	0.5872^*∗*^
mMRIA R1	0.5940^*∗*^	0.3637^*∗*^	0.5899^*∗*^
MRIA R2	0.6037^*∗*^	0.3708^*∗*^	0.6031^*∗*^
mMRIA R2	0.5931^*∗*^	0.3851^*∗*^	0.5920^*∗*^

^*∗*^ *p* < 0.0001.
